# An imaging and diagnostic conundrum—the adrenal haemangioma

**DOI:** 10.1093/jscr/rjae286

**Published:** 2024-05-03

**Authors:** Tiffany Tan, Jason Diab, Philip Chia, Amandeep Singh, Peter Campbell, Ronald Guevara

**Affiliations:** Department of Endocrine Surgery, Liverpool Hospital, Liverpool, NSW 2170, Australia; Department of Endocrine Surgery, Liverpool Hospital, Liverpool, NSW 2170, Australia; School of Medicine, University of New South Wales, Sydney, NSW 2052, Australia; School of Medicine, University of Notre Dame, Darlinghurst, NSW 2010, Australia; Department of Endocrine Surgery, Liverpool Hospital, Liverpool, NSW 2170, Australia; School of Medicine, University of Sydney, NSW 2052, Australia; Department of Endocrine Surgery, Liverpool Hospital, Liverpool, NSW 2170, Australia; School of Medicine, University of New South Wales, Sydney, NSW 2052, Australia; Department of Anatomical Pathology, Liverpool Hospital, Liverpool, NSW 2170, Australia; Department of Endocrine Surgery, Liverpool Hospital, Liverpool, NSW 2170, Australia; School of Medicine, University of Sydney, NSW 2052, Australia; Department of Endocrine Surgery, Liverpool Hospital, Liverpool, NSW 2170, Australia

**Keywords:** adrenal haemangioma, cavernous adrenal haemangioma, adrenalectomy, adrenal incidentaloma

## Abstract

The adrenal haemangioma, a rare benign vascular tumour, is increasingly detected through abdominal imaging. Just over 70 surgical cases have been reported since 1955. Their potential large size and overlapping imaging features with adrenocortical carcinoma poses a diagnostic challenge. Adrenalectomy is often needed for a definitive diagnosis due to inconclusive imaging. We report the case of a 61-year-old female presenting with an incidental finding of a right-sided 9.5-cm adrenal mass on imaging. Due to the risk of adrenocortical carcinoma with inconclusive imaging findings, an open right adrenalectomy was performed. The patient was discharged after 6 days with no complications. Post-surgical histopathology confirmed a diagnosis of adrenal haemangioma with a secondary adrenal pseudocyst. The presence of an adrenal incidentaloma with discordant radiological features proves to be a diagnostic conundrum. Therefore, in the setting of contradictory radiology and concerning mass size, we recommend adrenalectomy for definitive diagnosis of an adrenal haemangioma.

## Introduction

The adrenal haemangioma, also known as cavernous haemangioma, is a rare, benign vascular neoplasm of the adrenal gland. Patients may present without discernible symptoms and its incidental discovery has become more prevalent with widespread utilization of CT imaging. Since its first description in 1955, there have been approximately 74 surgical cases reported [1, 2]. It poses a diagnostic challenge due to discordant imaging findings, often warranting additional imaging and biochemical investigations to exclude malignancy or functional tumours. Adrenalectomy often remains imperative for definitive diagnosis and to minimize potential complications. This case report outlines the challenges in diagnosing an adrenal hemangioma through imaging and histopathology.

## Case report

**Figure 1:**
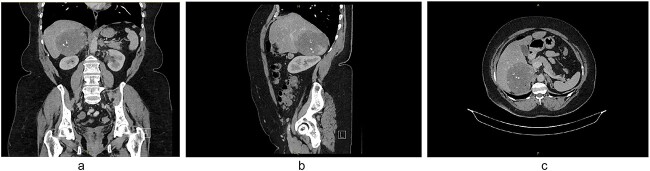
(a) CT coronal, (b) CT sagittal, (c) CT axial scans demonstrating a right-sided adrenal lesion (9.5 × 8.6 cm) with fat lobules, calcification and heterogeneous progressive enhancement.

A 61-year-old obese (BMI = 35.2) female of Maltese background attended her general practitioner with non-specific intermittent abdominal pain over 5 years. Her past medical history included hypertension and hypercholesterolaemia, and previous surgical history of hysterectomy and laparoscopic appendicectomy. She did not have any signs or symptoms suggestive of a functional adrenal mass. She denied significantly elevated blood pressure, flushing, syncope or palpitations. Clinically, she had no abdominal masses or regional tenderness on deep palpation. A CT scan demonstrated a right-sided adrenal lesion (9.5 × 8.6 cm) with fat lobules, calcification and heterogeneous progressive enhancement (Fig. 1).

She was referred to our institution and an adrenal biochemical workup showed normal results: potassium 4.7 mmol/L, plasma renin 6.6 mU/L, plasma aldosterone 228 pmol/L, aldosterone renin ratio 35, 1 mg dexamethasone suppression test 44 nmol/L, plasma normetanephrines 580 pmol/L, plasma metanephrines 150 pmol/L, DHEAS 2.3umol/L, chromogranin A 88ug/L.

**Figure 2:**
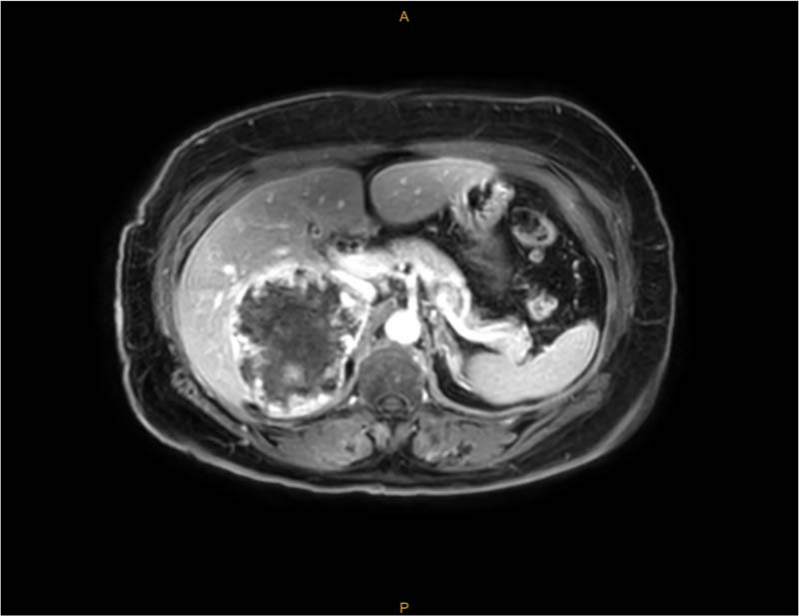
MRI—demonstrating peripheral enhancement in large, complex right-sided adrenal lesion (9.8 × 8.2 × 8.1 cm).

**Figure 3:**
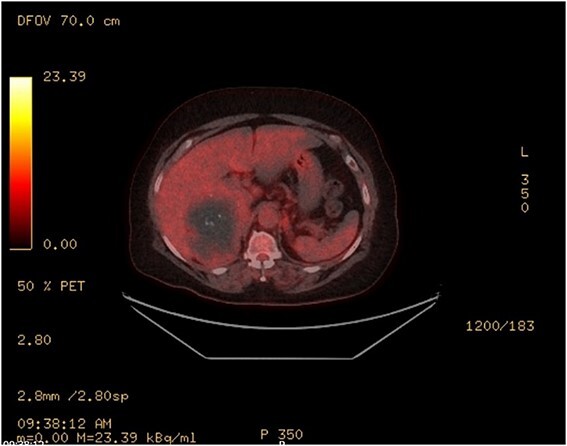
PET—large heterogenous complex right adrenal mass that measured up to 10 cm is largely photopenic with a mild peripheral rim of FDG accumulation (SUV max 3.4) at a level similar to adjacent physiologic hepatic activity.

An MRI scan with gadolinium was arranged, demonstrating a complex right adrenal mass (9.8 × 8.2 × 8.1 cm). There was heterogeneous T1 and T2 signal with nodular peripheral enhancement and progressive filling-in (Fig. 2), suggestive of a giant adrenal haemangioma. A PET scan further demonstrated a photopenic mass with a mild peripheral rim of FDG accumulation (SUV max 3.4) (Fig. 3). The appearances did not suggest high-grade malignancy. A red cell scan did not identify progressive accumulation on delayed imaging, atypical for a haemangioma.

**Figure 4:**
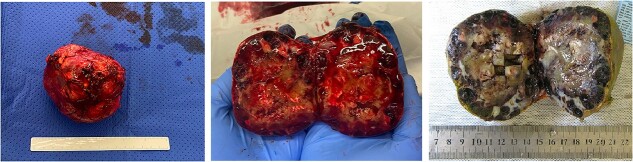
Macroscopic appearance with dilated blood-filled channels with tan-pale areas of fibrin, fibrosis, calcifications and haemorrhage (middle). With formalin fixation, these features are better appreciated (right).

**Figure 5:**
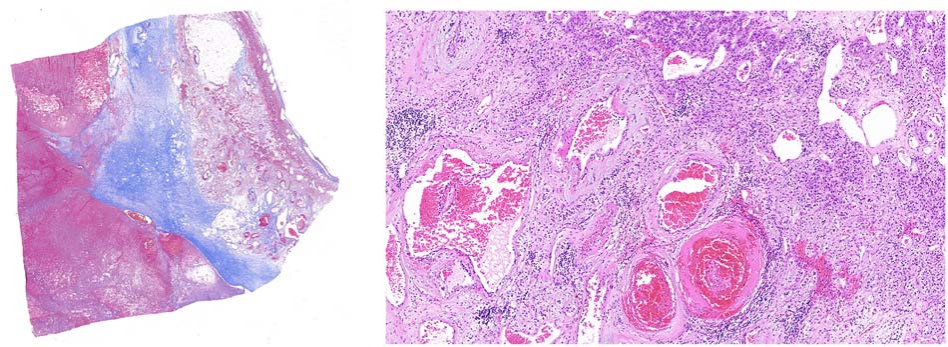
Left panel: low magnification (20×) of Masson’s trichrome stain highlighting fibrosis, fibrin and haemangioma in the adrenal cortex. Right panel: medium magnification (200×) of haematoxin- and eosin-stained section demonstrating variably dilated vascular changes filled with blood with variable hyalinization and fibrin in the wall.

The case was discussed at a multidisciplinary meeting with the decision to advocate for an open adrenalectomy given the size and radiological findings. Intraoperatively, the lesion was 10.3 × 8.5 × 6.9 cm (Fig. 4) and weighed 319 g. Histologically, salient features of a pseudocyst were appreciated including extensive stromal fibrosis, fibrin deposition, dystrophic calcifications and areas of hyalinization, suggesting repeated previous haemorrhage and reorganization from an underlying haemangioma (Fig. 5). There were no features suggestive of a malignancy. The patient was discharged 6 days post-operatively and was well at the 8-week follow-up.

## Discussion

Cavernous haemangiomas are uncommon vascular tumours showing a predilection for skin, liver or brain [3]. Adrenal haemangiomas are rarer with an unclear pathogenesis, presenting a diagnostic challenge due to radiological features often resembling adrenocortical carcinoma [4]. Only 74 cases were reported from 1955 to 2021 and the median age was 61.7 years, with a slight female preponderance (female:male, 1.5:1) [2]. Most adrenal haemangiomas are found incidentally and patients tend to be asymptomatic [5], unless they have a large palpable mass or hypovolaemic shock secondary to spontaneous lesion rupture [6].

Radiologically, adrenal haemangiomas are reported as a hypodense, heterogenous lesion with speckled calcifications on CT scans [3, 7]. Concerns for adrenocortical carcinoma arise with findings of calcifications, necrosis and haemorrhage—especially in masses exceeding 4 cm [5]. On MRI, haemangiomas commonly present with densely enhanced peripheral rims and patchy enhancement; however, they may also present as T1 hypointense and T2 intermediate-high signal with varying enhancement [3, 8]. PET scans have a high utility for further characterization with uptake at a physiologic level below reference liver activity being consistent with a benign lesion [7, 9]. Red cell scans are a useful adjunct to characterize bleeding sites, commonly used to detect liver hemangiomas. Findings demonstrate diminished perfusion with delayed filling, known as perfusion blood-pool mismatch, a phenomenon attributed to delayed blood flow within the haemangioma compared to surrounding normal tissue [7, 10]. Unfortunately, red cell scans have not yet been investigated for their utility with adrenal hemangiomas. Our case demonstrated features of a heterogeneous, calcified mass on CT suggestive of malignancy, but subsequent MRI and PET had peripheral enhancement with central progressive filling, and a lack of hypermetabolic activity, respectively. Interestingly, our patient’s red cell scan reported atypical features of haemangioma, specifically the lack of perfusion blood pool mismatch, contradicting MRI findings. Therefore, in the setting of discordant radiology and size, we recommend adrenalectomy for subsequent pathology.

Histologically, adrenal haemangiomas can be subtyped into cavernous, capillary, venous and mixed [11]. Cavernous haemangiomas have well-defined margins and are usually confined to the adrenal cortex. They exhibit numerous dilated vascular channels lined by bland endothelium, and with repeated haemorrhage can show complex necrotic, calcified, fibrotic and thrombotic changes, features compatible with reorganization (‘pseudocyst’) [12]. In our case, these features were well evident (Fig. 5).

A multidisciplinary approach is fundamental to the workup of adrenal lesions, particularly in cases with inconclusive radiological findings. Notably, the recommendation for excision of adrenal incidentalomas >6 cm in diameter stems from the substantial risk (35–98%) of adrenal cancer in lesions of this size [13]. This is reinforced by frequent observation of calcifications in malignant adrenal cortical tumours, with an incidence rate of 31% [14]. Conversely, an alternative perspective suggests adrenalectomy for lesions >4 cm [4, 5], introducing variability in clinical decision making.

In the absence of other concerning radiological findings, homogenous adrenal lesions <4 cm, featuring a lipid-rich core (<10 HU) are consistent with benign adrenal lesions. These can be managed conservatively with observation and regular follow-up imaging [15]. Almost all case reports of adrenal haemangiomas were treated surgically with their diagnosis commonly made following histology. Resection serves not only as a diagnostic measure but also proves therapeutic in instances of larger hemangiomas, alleviating compression exerted by the lesion on adjacent organs. Albeit rare, larger lesions can carry the risk of potential spontaneous haemorrhage [2]. In the setting of possible malignancy with discordant imaging, we recommend surgical resection in the presence of a functioning mass, increasing lesion size with or without mass effect for exclusion of malignant pathology, or haemorrhagic complications.

## Conclusion

Adrenal haemangiomas are rare benign tumours warranting consideration as a differential diagnosis in the evaluation of adrenal incidentalomas. Radiological concordance and biochemical workup are the cornerstone towards a diagnosis whilst excluding malignancy. Adrenalectomy should be considered for lesions with increasing size, mass effect or concern of malignant potential.

## References

[1] Johnson CC , Jeppesen FB. Hemangioma of the adrenal. J Urol 1955;74:573–7. 13272266 10.1016/S0022-5347(17)67320-8

[2] Chua Y , Quake S, Prasad K, Elsaify W. A rare case of cavernous haemangioma of the adrenal gland: a case report and review of literature. Cureus 2022;14:e29917. 36348862 10.7759/cureus.29917 PMC9633059

[3] Dunnick NR , Korobkin M, Francis I. Adrenal radiology: distinguishing benign from malignant adrenal masses. AJR Am J Roentgenol 1996;167:861–7. 8819372 10.2214/ajr.167.4.8819372

[4] Nishtala M , Cai D, Baughman W, McHenry CR. Adrenal cavernous hemangioma: a rare tumor that mimics adrenal cortical carcinoma. Surg Open Sci 2019;1:7–13. 32754687 10.1016/j.sopen.2019.04.001 PMC7391906

[5] Antar RM , Farag CM, Youssef K, et al. Rare adrenal cavernous hemangioma: a case report highlighting diagnostic challenges. Front Surg 2023;10:1293925. 38026486 10.3389/fsurg.2023.1293925 PMC10667707

[6] Peng X , Luo W, Zhang X, Zhu W. Sudden onset flank pain: a case report of retroperitoneal hemorrhage secondary to a ruptured adrenal hemangioma. J Pain Res 2018;11:1421–4. 30104897 10.2147/JPR.S160661 PMC6074836

[7] Ploussard B , Kieffer A, Ramasamy SK, et al. Unusual presentation of adrenal hemangioma as an incidental large adrenal hematoma - a case report. Clin Imaging 2022;84:61–4. 35149234 10.1016/j.clinimag.2022.01.007

[8] Huang T , Yang Q, Hu Y, Wu HX. Adrenal cavernous hemangioma misdiagnosed as pheochromocytoma: a case report. BMC Surg 2021;21:210. 33902538 10.1186/s12893-021-01195-2 PMC8074472

[9] Wilson B , Becker A, Estes T, et al. Adrenal hemangioma definite diagnosis on CT, MRI, and FDG PET in a patient with primary lung cancer. Clin Nucl Med 2018;43:e192–4. 29561525 10.1097/RLU.0000000000002069

[10] Brodsky RI , Friedman AC, Maurer AH, et al. Hepatic cavernous hemangioma: diagnosis with 99mTc-labeled red cells and single-photon emission CT. AJR Am J Roentgenol 1987;148:125–9. 3491500 10.2214/ajr.148.1.125

[11] Kansoun A , El-Helou E, Mazraani HB, et al. Adrenal hemangioma: a rare presentation of bleeding incidentaloma: case report. Int J Surg Case Rep 2020;77:442–5. 33395822 10.1016/j.ijscr.2020.11.024 PMC7691671

[12] Tarchouli M , Boudhas A, Ratbi MB, et al. Giant adrenal hemangioma: unusual cause of huge abdominal mass. Can Urol Assoc J 2015;9:E834–6. 26600897 10.5489/cuaj.2967 PMC4639440

[13] Aljabri KS , Bokhari SA, Alkeraithi M. Adrenal hemangioma in a 19-year-old female. Ann Saudi Med 2011;31:421–3. 21293064 10.4103/0256-4947.76411 PMC3156522

[14] Rothberg M , Bastidas J, Mattey WE, Bernas E. Adrenal hemangiomas: angiographic appearance of a rare tumor. Radiology 1978;126:341–4. 622479 10.1148/126.2.341

[15] Fassnacht M , Tsagarakis S, Terzolo M, et al. European Society of Endocrinology clinical practice guidelines on the management of adrenal incidentalomas, in collaboration with the European Network for the Study of Adrenal Tumors. Eur J Endocrinol 2023;189:G1–G42. 37318239 10.1093/ejendo/lvad066

